# The *AIB1 *glutamine repeat polymorphism is not associated with risk of breast cancer before age 40 years in Australian women

**DOI:** 10.1186/bcr1009

**Published:** 2005-03-04

**Authors:** Karen G Montgomery, Jiun-Horng Chang, Dorota M Gertig, Gillian S Dite, Margaret R McCredie, Graham G Giles, Melissa C Southey, John L Hopper, Ian G Campbell

**Affiliations:** 1 Cancer Genetics Laboratory, Victorian Breast Cancer Research Consortium, Peter MacCallum Cancer Centre, St. Andrews Place, East Melbourne, Victoria, Australia; 2 Centre for Genetic Epidemiology, The University of Melbourne, Carlton, Victoria, Australia; 3 Department of Preventive and Social Medicine, University of Otago, Dunedin, New Zealand and (previously) Cancer Epidemiology Research Unit, The Cancer Council of New South Wales, Sydney, New South Wales, Australia; 4 Cancer Epidemiology Centre, The Cancer Council Victoria, Carlton, Victoria, Australia; 5 Department of Pathology, The University of Melbourne, Parkville, Victoria, Australia

## Abstract

**Introduction:**

*AIB1*, located at 20q12, is a member of the steroid hormone coactivator family. It contains a glutamine repeat (CAG/CAA) polymorphism at its carboxyl-terminal region that may alter the transcriptional activation of the receptor and affect susceptibility to breast cancer through altered sensitivity to hormones.

**Methods:**

We evaluated this repeat polymorphism in the context of early-onset disease by conducting a case-control study of 432 Australian women diagnosed with breast cancer before the age of 40 years and 393 population-based control individuals who were frequency matched for age. Genotyping was performed using a scanning laser fluorescence imager.

**Results:**

There were no differences in genotype frequencies between cases and control individuals, or between cases categorized by family history or by *BRCA1 *and *BRCA2 *germline mutation status. There was no evidence that the presence of one or two alleles of 26 glutamine repeats or fewer was associated with breast cancer (odds ratio = 1.03, 95% confidence interval = 0.73–1.44), or that women with alleles greater than 29 repeats were at increased risk of breast cancer. Exclusion of women who carried a *BRCA1 *or *BRCA2 *mutation (24 cases) and non-Caucasian women (44 cases) did not alter the risk estimates or inferences. We present raw data, including that on mutation carriers, to allow pooling with other studies.

**Conclusion:**

There was no evidence that risk of breast cancer depends on *AIB1 *CAG/CAA polymorphism status, even if affected women carry a mutation in *BRCA1 *or *BRCA2*.

## Introduction

Steroid hormones regulate the expression of proteins that are involved in breast cell proliferation and development, and coactivators that interact with steroid hormone receptors to modulate transcriptional activation have recently been described [1]. *AIB1*, located at 20q12, is a member of the steroid hormone coactivator family that interacts with oestrogen receptor-α, resulting in enhancement of oestrogen-dependent transcription [2]. *AIB1 *is moderately expressed in the normal mammary epithelium and is required for female reproductive function and mammary gland development [3]. It is overexpressed in 64% of breast tumours, and the 20q12 region has been shown to be amplified in 5–10% of breast cancers and 7% of ovarian cancers [2,4].

*AIB1 *contains a glutamine repeat (CAG/CAA) polymorphism at its carboxyl-terminal region. Although its functional significance is currently unknown, it may alter the transcriptional activation of the receptor (as is the case for the androgen receptor CAG repeat polymorphism), and hence it may affect breast cancer susceptibility through altered sensitivity to hormones [5]. We evaluated this repeat polymorphism within the context of early-onset disease by conducting a case-control study of Australian women diagnosed with breast cancer before the age of 40 years and population-based control individauls [6,7].

## Materials and methods

### Participants

The Australian Breast Cancer Family Study is a population-based, case-control family study conducted in Melbourne and Sydney [6,8]. For this study, cases were women aged under 40 years at diagnosis of a first primary invasive breast cancer between 1992 and 1995, and were identified through the Victoria and New South Wales cancer registries. Controls were women without breast cancer selected via the electoral rolls (registration is compulsory) between 1993 and 1999 and were frequency matched for age. Cases and controls were administered the same questionnaire on risk factors, blood samples were collected from them at the time of interview, and a detailed family history was recorded for all first-degree and second-degree relatives, with verification sought for all reports of family cancers. To date 25 of these cases have been found to carry a deleterious germline mutation in *BRCA1 *and 11 in *BRCA2 *[9]. Written informed consent was obtained from all participants, and approval of the protocol was obtained from the relevant ethics committees.

### Genotype analysis

PCR amplifications were performed with a fluorescent labelled forward primer (5'-GACAACAGAGGGTGGCTAT-3') and an unlabelled reverse primer (5'-AGGAGCTTGTGGCATTGTG-3'). All PCRs were performed in 10 μl volumes containing 10–50 ng genomic DNA, 200 nmol/l dNTPs (Promega, Annandale, New South Wales, Australia), 25 ng of each primer, 1 × ReddyMix buffer (Abgene, Epsom, Surrey, UK) and 0.2 units of Thermoprime Plus DNA Polymerase (Abgene). PCR amplification cycle conditions involved an initial denaturation step at 94°C for 5 min, 40 cycles of denaturation at 94°C for 15 s, annealing at 55°C for 30 s, and extension at 72°C for 45 s. This was followed by a further extension step at 72°C for 7 min. The alleles were then separated on sequencing gels and analyzed using a scanning laser fluorescence imager (Bio-Rad FX Molecular Imager; Bio-Rad, Hercules, CA, USA). DNA was available for 410 (88%) out of 466 cases and for 441 (74%) out of 600 controls, and *AIBI *genotyping was successful for all but 17 (4%) cases and 9 (2%) controls.

### Statistical analysis

Allele frequencies and genotypes were compared using Pearson's χ^2 ^test. Odds ratios (ORs) and 95% confidence intervals (CIs) were calculated using unconditional logistic regression, adjusting for reference age, study centre, country of birth, education level, marital status, number of live births, height, current oral contraceptive use status and reported first-degree family history. Routine model diagnostics procedures and goodness-of-fit were performed to check the adequacy of the logistic regression models. All tests were performed using Stata version 8.0 (Stata Corporation, College Station, TX, USA). Power calculations were performed using StatCalc module of Epi Info version 6 (Centers for Disease Control and Prevention [CDC], Atlanta, GA, USA). All statistical tests were two-sided.

## Results

For controls the allele frequencies for 26, 27, 28, 29, 30 and 31 repeats were 0.12, 0.002, 0.36, 0.50, 0.005 and 0.002, respectively. For cases they were 0.13, 0.001, 0.35, 0.51, 0.004 and 0, respectively. Table 1 shows that there was no difference overall between breast cancer cases and controls in allele frequencies (*P *= 0.7) or genotype frequencies defined by the number of repeats (*P *= 0.3). There were no differences in the genotype distribution of cases by family history (*P *= 0.1).

Table 2 shows the estimates of breast cancer risk; we chose a cutoff of 26 repeats or fewer because that had been used in a previous study [1]. There was no evidence that women with one or two alleles had increased risk of breast cancer (OR = 1.03, 95% CI = 0.73–1.44) or that women with alleles of greater than 29 repeats were at increased risk. Exclusion of women who carried a *BRCA1 *or *BRCA2 *mutation (24 cases) and non-Caucasian women (44 cases) did not alter risk estimates or inferences. For example, the OR for the association with one or two alleles of 26 repeats or fewer became 1.09 (95% CI = 0.76–1.56).

## Discussion

In the present study of Australian women the allele frequencies and genotype distributions for the *AIB1 *glutamine repeat (CAG/CAA) polymorphism were similar to those reported in previous studies [1,7]. We found no evidence that the risk of early-onset breast cancer depended on *AIB1 *genotype. We have presented the raw data, including that on mutation carriers, to allow pooling with other studies.

The only other study of breast cancer risk and this polymorphism in noncarriers of a *BRCA1 *or *BRCA2 *mutation was conducted in women aged 43–69 years [1]; it found no association. Those investigators found a weak suggestion that premenopausal women who carried two short *AIB1 *repeats were at decreased risk, but we did not find any support for this in women under the age of 40 years.

Evidence for an increased risk in women with longer repeat lengths has been reported in *BRCA1 *and *BRCA2 *mutation carriers [3]. There was no indication from our data that mutation carriers, irrespective of the gene, were at increased risk if they carried longer repeat lengths, but because of the small numbers included we had little power to address this issue.

## Conclusion

We found no evidence that risk of breast cancer depends on *AIB1 *CAG/CAA polymorphism status, even if affected women carry a mutation in *BRCA1 *or *BRCA2*.

## Abbreviations

CI = confidence interval; OR = odds ratio; PCR = polymerase chain reaction.

## Competing interests

The author(s) declare that they have no competing interests.

## Authors' contributions

KGM and IGC conceived the study; participated in its design, concept and coordination; and drafted the manuscript. J-HC performed the statistical analysis. DMG, JLH, GGG and MRM participated in the concept and coordination, are the chief investigators and developed ABCFS, and helped to draft the manuscript. GSD contributed to the design and management of data. MCS contributed to the design and management of laboratory processes at the GEL to enable and provide the required biospecimens for genetic analysis.

## Figures and Tables

**Table 1:** Distribution of genotypes defined by observed *AIB1 *glutamine repeat alleles

Number of repeats in AIB1	Controls	Cases	Total	Cases			
				
				Without family history	With family history	*BRCA1 *mutation carriers	*BRCA2 *mutation carriers
26/26	5 (1.2)	9 (2.3)	14 (1.7)	4 (1.5)	5 (4.3)	0 (0)	0 (0)
26/28	39 (9.0)	34 (8.7)	73 (8.9)	24 (8.7)	10 (8.6)	2 (14.3)	0 (0)
26/29	58 (13.4)	52 (13.2)	110 (13.3)	40 (14.5)	12 (10.3)	0 (0)	1 (9.0)
27/28	1 (0.2)	0 (0)	1 (0.1)	0 (0)	0 (0)	0 (0)	0 (0)
27/29	1 (0.2)	1 (0.3)	2 (0.2)	1 (0.36)	0 (0)	0 (0)	0 (0)
28/28	56 (13.0)	64 (16.3)	120 (14.6)	52 (18.8)	12 (10.3)	5 (35.7)	2 (18.2)
28/29	160 (37.0)	116 (29.5)	276 (33.5)	81 (29.4)	35 (29.9)	3 (21.4)	3 (27.3)
28/30	2 (0.5)	1 (0.3)	3 (0.4)	1 (0.34	0 (0)	0 (0)	0 (0)
29/29	106 (24.5)	114 (29.0)	220 (26.7)	71 (25.7)	43 (36.8)	4 (28.6)^a^	5 (45.5)^a^
29/30	2 (0.5)	2 (0.5)	4 (0.5)	2 (0.7)	0 (0)	0 (0)	0 (0)
29/31	2 (0.50)	0 (0)	2 (0.2)	0 (0)	0 (0)	0 (0)	0 (0)
Total	432	393	825	276	117	14	11

Shown is the distribution of genotypes defined by the observed *AIB1 *glutamine repeat alleles (*n *[%]) in cases and controls, and in cases categorized by family history and by *BRCA1 *and *BRCA2 *germline mutation status. ^a^One case, with the 29/29 repeats *AIB1 *genotype, had a germine mutation in *BRCA1 *and a germline mutation in *BRCA2 *[9].

**Table 2:** *AIB1 *genotype and risk of breast cancer

Genotype^a^	Cases (*n *[%])	Controls (*n *[%])	OR (95% CI)^b^
0	298 (76)	330 (76)	1.00 (reference)
1	86 (22)	97 (23)	0.97 (0.68–1.38)
2	9 (2)	5 (1)	2.17 (0.69–6.85)
1 or 2	95 (24)	102 (24)	1.03 (0.73–1.44)

^a^Genotype defined as number of alleles with 26 or fewer repeats. ^b^Adjusted for study centre (Melbourne/Sydney), reference age (years), country of birth (Australia/other), education level (three levels), marital status (ever/never), number of live births, height (cm), current oral contraceptive use (yes/no) and affected first-degree relative (yes/no). CI, confidence interval; OR, odds ratio.
